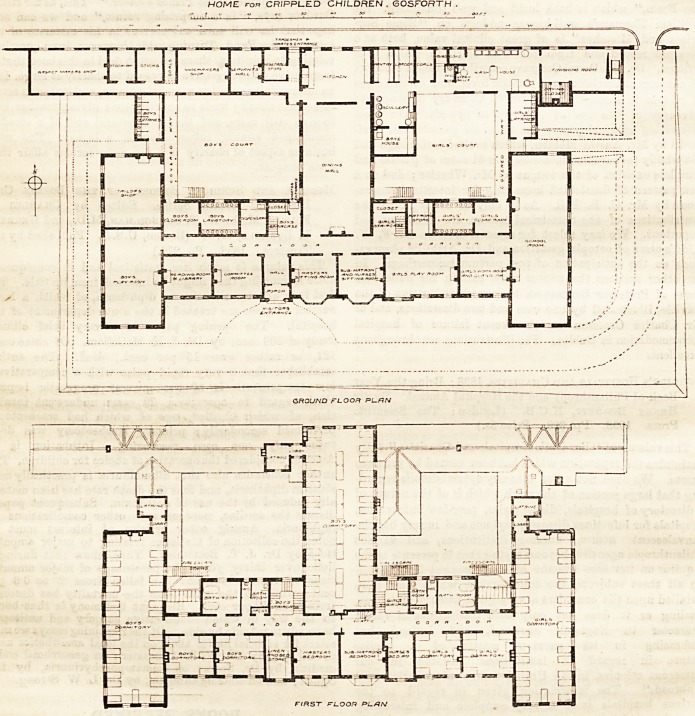# Hospital Construction

**Published:** 1898-07-02

**Authors:** 


### The Institutional Workshop.

### HOSPITAL CONSTRUCTION.

### HOME FOR CRIPPLED CHILDREN, GOSFORTH, NEWCASTLE-ON-TYNE.

The erection of this home is an excellent example of the growth of a deserving and well-managed charity from very small beginnings to a large establishment. Only ten years ago the founders began their work by taking care of six poor crippled children, and the house they then rented has now been succeeded by the building illustrated, accommodating 110 (73 boys and 37 girls). As will be seen from the plans, the building has a central block, and two wings at right angles to it, the entrance being to the west of the centre. The wings contain, on the ground floor, the boys' play-room and tailor's shop on the west, and the large mixed school-room (60 ft. by 20 ft.) on the east; while between them are the reading-room, committee-room, master's and matron's sitting-rooms, and girls' play and work rooms, all facing south, with lavatories, dispensary, cloak-rooms, and the large dining-hall behind. Beyond the latter is a range of one-storied buildings, containing the kitchen, pantries, &c., basket and shoe making shops for the boys, and a laundry for the girls. The w.c.'s for each sex are between this range and the main wings, and are connected to the other buildings by open corridors. There are two staircases, leading to the three large dormitories, one, for girls, over the school-room, and two, for boys, over the dining-hall and west wing. Each of these have lavatory accommodation, properly disconnected by ventilating lobbies, and there are also two small dormitories for each sex, with bed-rooms for the staff, in the front of the main building, and bath-rooms at the back, next the stairs. There are wings, and are connected to the other buildings by open corridors.

four baths for the children, and two for the staff, and the latter have bedrooms on the second floor, besides those below.

The general dispositions of the plan seem suitable and satisfactory, and aspect has been well considered. It is, however, difficult to see how the ground-floor corridor can have any light or ventilation other than that coming from the two windows of the staircases, and the two main lavatories must also be under-lighted, while the ground-floor closets are rather more hemmed in by buildings than is desirable. On the first floor the corridor is lighted at each end, and the closets are suitably placed, but some of the dormitories seem unduly crowded, the floor space in the largest being only 40 ft. per bed, and one may hope the central row of beds may be found unnecessary. The architect is Mr. E. Shewbrooks, of Newcastle.

## Figures and Tables

**Figure f1:**